# A Single-Center Experience of Dopamine Antagonist ONC201 for Recurrent Histone H3 Lysine 27-to-Methionine (H3K27M)-Mutant Glioblastoma in Adults

**DOI:** 10.7759/cureus.28175

**Published:** 2022-08-19

**Authors:** Chukwuyem Ekhator, Ramin Rak, Ramya Tadipatri, Ekokobe Fonkem, Jai Grewal

**Affiliations:** 1 Neuro-Oncology, New York Institute of Technology College of Osteopathic Medicine, Old Westbury, USA; 2 Neurosurgery, Neurological Surgery, PC (NSPC) Rockville Center, New York City, USA; 3 Neurology, Barrow Neurological Institute, Phoenix, USA; 4 Neuro-Oncology, Baylor Scott and White Medical Center, Temple, USA; 5 Neuro-Oncology, Mount Sinai South Nassau Hospital, Oceanside, USA

**Keywords:** md anderson symptom inventory instrument (mdasi), karnofsky performance scale (kps), h3k27m mutation, onc018, glioblastoma, onc-201

## Abstract

This study aimed to report a single-center experience of three adult subjects receiving ONC201 as part of the ONC018-expanded access clinical trial (NCT03134131). ONC201 is an oral investigational antagonist against the D2 dopamine receptor that has shown encouraging results for malignant gliomas harboring the histone H3 lysine 27-to-methionine (H3K27M) mutation in the H3 histone complex. Responses have been reported in pediatric subjects with such tumors. An expanded access clinical trial (ONC018) was available to eligible patients allowing them access to this agent pending FDA review. Our site enrolled three subjects in the ONC018 trial. We present the demographic, clinical, and molecular characteristics of our enrolled subjects. We report the tolerability, adverse events, and outcome measures including survival, Karnofsky Performance Status (KPS), and quality-of-life measured by the MD Anderson symptom inventory instrument (MDASI). Three subjects were registered at our site onto ONC018 with the age range of 18-44 years, two of three were female, residing in Norway, India, and the United States. Tumor locations were brainstem, corpus callosum, and thalamus. Pathology includes glioblastoma (3/3), methylguanine-DNA methyltransferase (MGMT) methylated (2/3), isocitrate dehydrogenase 1 (IDH1) mutant (0/3), epidermal growth factor receptor (EGFR) amplification (0/3), and α thalassemia/mental retardation syndrome X‑linked (ATRX) (3/3). Median change from baseline KPS ≤20% decrease; MDASI of 2/3 experienced decrease from baseline (median 6%), consistent with improved quality of life. No clinically significant laboratory abnormalities were found. All adverse events were grades I-II. We found that the study drug was quite tolerable. No serious adverse events nor radiographic responses were seen. Analyses of the larger study cohort and additional randomized controlled trials are necessary to provide insight into the safety and efficacy.

## Introduction

ONC201 is a small molecule, orally active having heterocyclic pharmacophore with favorable efficacy and safety studies, and it is an antagonist to the G-protein coupled dopamine receptor D2 [1]. ONC201 is available as a novel anti-tumor, investigational drug product in phase two of drug development process while phase one study results have shown that it reaches faster micromolar values in plasma and has a favorable tolerability profile in cancer patients [2-5]. ONC201 is an imipridone that is used in multiple cancer forms, and it produces integrated stress response by downstream activation in tumor cells [1,6]. Further powerful effects of ONC201 include prevention of proliferation of cancerous cells and apoptosis-like effects against a broad variety of tumors [3,5]. The advantage of ONC201 is the fact that it does not affect normal cells [7,3].

Gene expression analysis demonstrates that the pathological mechanisms undergo various molecular disturbances leading to induction of stress mechanism either by integrated stress response (ISR) or by endoplasmic reticulum stress (ER). The activation of stress mechanism leads to disturbance of cell normal homeostasis by accumulation of misfolded or disturbed proteins, the response also known as unfolded protein response (UPR) [8-11]. Both pathways may lead to programmed cell death process known as apoptosis at peak protein folding disturbance. This proposed mechanism is also termed atypical ISR [11-13]. ONC201 causes upregulation of various subsets of genes which ultimately result in upregulation process of tumor necrosis factor group of ligands (TRAIL) expression, especially in the case of cancer cell lines of various solid tumors. This mechanism of inactivation of genes subset was studied in two different studies being upregulation as a common contributor. Both studies present cytotoxic effect of ONC201 in TRAIL independent manner. ONC201 treatment resulted in the inactivation of extracellular signal-regulated kinase (ERK) and Akt in one study [6] while cyclophosphamide, doxorubicin, vincristine, and prednisone (CHOP) and activating transcription factor 4 (ATF4) in another study; both ATF4 and CHOP [1] were considered responsible for the cytotoxic effect of ONC201 in cancerous tumors [14].

Glioblastoma is a common, aggressive, and highly malignant brain tumor that may further be divided into primary or secondary glioblastoma and affects usually elderly patients more with high development incidence (90%). Glioblastomas are around 46.1% of primary malignant brain tumors [15]. Primary glioblastoma is characterized as without any histological or clinical evidence of previous lesion while the development of secondary glioblastoma develops from anaplastic astrocytoma or astrocytoma [16]. Pediatric gliomas are distinct from adult gliomas in terms of clinical and biological presentations. Adult gliomas progress from any less malignant precursor while pediatric gliomas are like secondary glioblastomas [17]. Demographic and gender-based data show that males are 1.6 times more prone to be affected by such brain tumors than females while incidence is two times more in white than black ones [18].

Histone H3 lysine 27 (H3K27)-mutant diffuse midline glioma is the most frequent histone mutation in gliomas [19]. It is defined as a grade IV disease in the 2016 WHO classification [20]; such mutations are more common in adults and especially in the brain’s midline (high ratio of 50-90% of midline gliomas) [21]. No effective therapy was observed after first-line radiation therapy was seen [22]. Mutations in histone H3 complex in high-grade glioma in pediatrics may result in changes in epigenetic genes which ultimately induce changes in the gene transcription process [23]. The histone H3 lysine 27 (H3K27) is most influential in cancer genes as it is responsible for the transcription process [24,25]. The amount of methylation on H3K27 is the main contributor to DNA functional properties, hence methylation is directly correlated with the activity of the enhancer of zeste homolog 2 (EZH2) methyltransferase which is considered active and therapeutic target in various human cancers [26-28].

Given the important role that metabolism plays in the inflammatory and functional condition of macrophages, as well as the metabolic alterations that occur in tumor cells, metabolic reprogramming is an incredibly appealing therapeutic option. In recent years, ONC201 has been shown to be a highly beneficial medicine in the treatment of cancer, and it is now being tested in clinical studies for glioblastoma. In glioblastoma, ONC201 has the ability to exert its action not only by targeting tumor cells but also by causing a metabolic transition in tumor-associated microphages. This would activate their pro-inflammatory actions, which would assist and/or enhance the cytotoxicity of ONC201 against tumor cells.

We report a single-center experience of three adult subjects receiving ONC201 as part of the ONC018-expanded access clinical trial (NCT03134131). In this study, we present three cases of glioblastoma with varying tumor locations which includes thalamus, brainstem, and corpus callosum. All three patients were from different geographical regions, and they were included in the ONC018 trial.

Our site enrolled three subjects in the ONC018 trial. We present the demographic, clinical, and molecular characteristics of our enrolled subjects. We report the tolerability, adverse events, and outcome measures including survival, Karnofsky Performance Status (KPS), and quality-of-life measured by the MD Anderson symptom inventory instrument (MDASI). Informed consent was obtained from all the patients. The ONC018 study was approved by the Institutional Review Board of the Mount Sinai South Nassau, New York, with WIRB protocol #20182894.

This article was previously presented as a meeting abstract at the 2022 American Academy of Neurology (AAN) Annual Scientific Meeting on April 3, 2022, and abstracted as follows: Ekhator C, Rak R, Tadipatri R, Fonkem E, Grewal J: CLRM-02 single center experience of dopamine antagonist ONC-201 for recurrent H3K27M-mutant glioblastoma in adults. Neuro-Oncology Advances. 2022, 4:i6. DOI: https://doi.org/10.1093/noajnl/vdac078.023.

## Case presentation

Case 1

An 18-year-old Norwegian male developed progressive back pain and paresthesia starting in 2017. He underwent imaging as the symptoms worsened and he was found to have a thoracic spinal mass. He underwent surgical resection after six months of symptom onset and was diagnosed with a WHO grade 1 ganglioglioma. He subsequently developed muscle strength, and his ability to walk, and developed bowel and bladder incontinence. He then underwent a second surgical operation after eight months, and his diagnosis was updated to a high-grade WHO 3/4 glioma with the presence of the H3K27M point mutation. He underwent radiation to the thoracic spine tumor for about one month, receiving 1.8 Gy x 28 fractions, and molecular profile was ordered from the second surgical specimen.

He received one cycle of the combination of temozolomide and lomustine (CCNU) as initial therapy after radiation therapy, and after one year, molecular report became available and showed the presence of FGFR1 and NF1 mutations. CCNU was discontinued and sorafenib monotherapy was initiated at 400 mg PO twice daily, and the patient experienced clinical and radiographic benefits for approximately six months. He had placement of a programmable ventriculoperitoneal shunt due to hydrocephalus. After that, he was started with ONC201 on weekly bases with five doses of 25 mg each.

**Figure 1:**
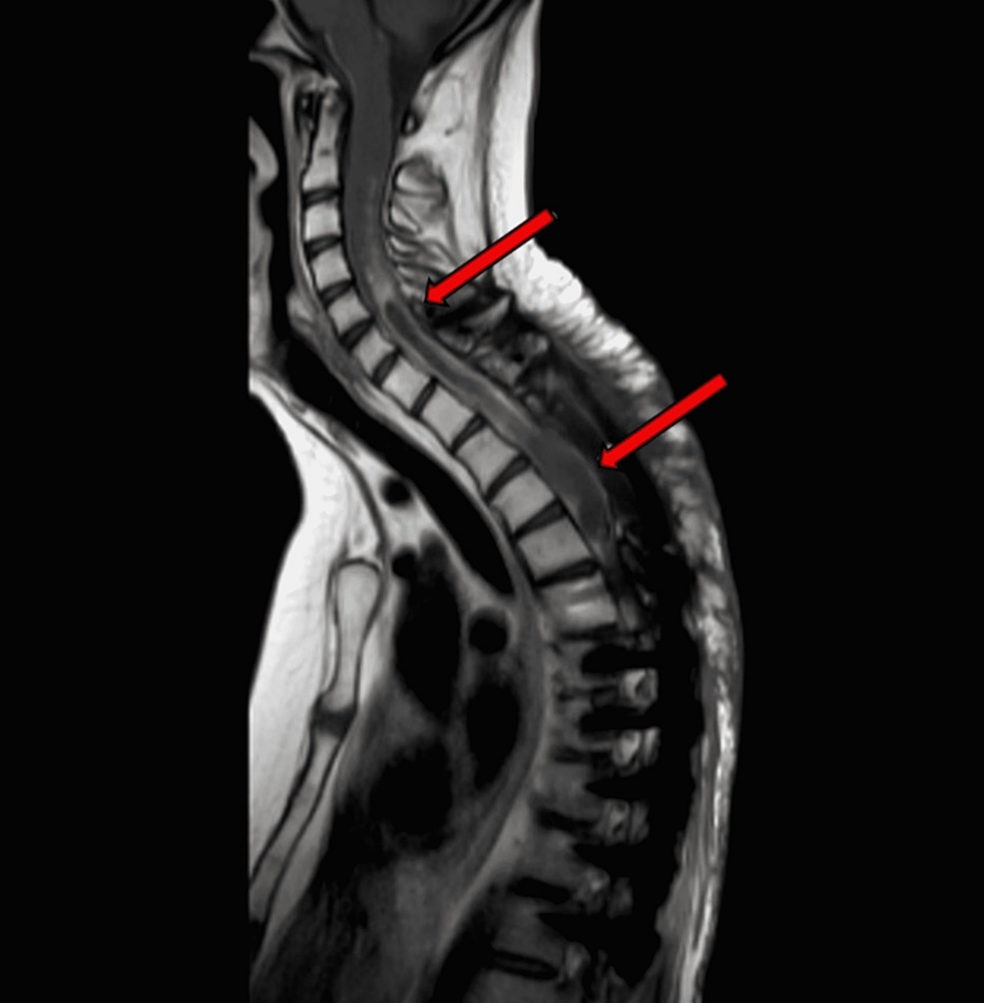
Sagittal MRI of thoracic spine showing decreased signal within the parenchyma of the upper cervical and thoracic cord (arrows)

Upon review of the cervical spine MRI performed, there appears to be decreased signal on T2 within the parenchyma of the upper cervical cord and brainstem (Figure 1). At this time, brain thoracic and lumbar spinal imaging were also made available, and it was noted that there is dissemination with tumor in the left anterior septum pellucidum as well as occipital horn area (Figure 1). It was also noted that there was stability of those brain lesions. With respect to enhancing disease, per Response Assessment in Neuro-Oncology (RANO) criteria, the assessment came under the category of stable disease. Characteristics of cases in this study are presented in Table 1. 

**Table 1:** Summary table outlining the tumor characteristics, location, genetic profile, and outcome of all three subjects MGMT: methylguanine-DNA methyltransferase; IDH1: isocitrate dehydrogenase 1; EGFR: epidermal growth factor receptor; ATRX: α thalassemia/mental retardation syndrome X‑linked, KPS: Karnofsky Performance Scale; MDASI: MD Anderson Symptom Inventory; OS: overall survival; PFS: progression-free survival

Summary	Case 1	Case 2	Case 3
Primary tumor location	Upper thoracic cord	Corpus callosum	Left thalamus
MGMT	Unmethylated	Methylated	Methylated
IDH1 status	Negative	Negative	Negative
EGFR status	Not amplified	Not amplified	Not amplified
ATRX	Detected	Detected	Detected
KPS median change from baseline	≤ 20% decline from baseline		
MDASI	Median 6% decrease from baseline		
ONC201-dosing and dispositions	Oral 625 mg ONC201. Once every week for 10 weeks		
Serious adverse events	This study (Table 2)		
RANO high-grade glioma criteria	Stable disease		
Median OS	7 months (28 weeks)		
PFS rate at 6 months	33%		

Case 2

**Figure 2:**
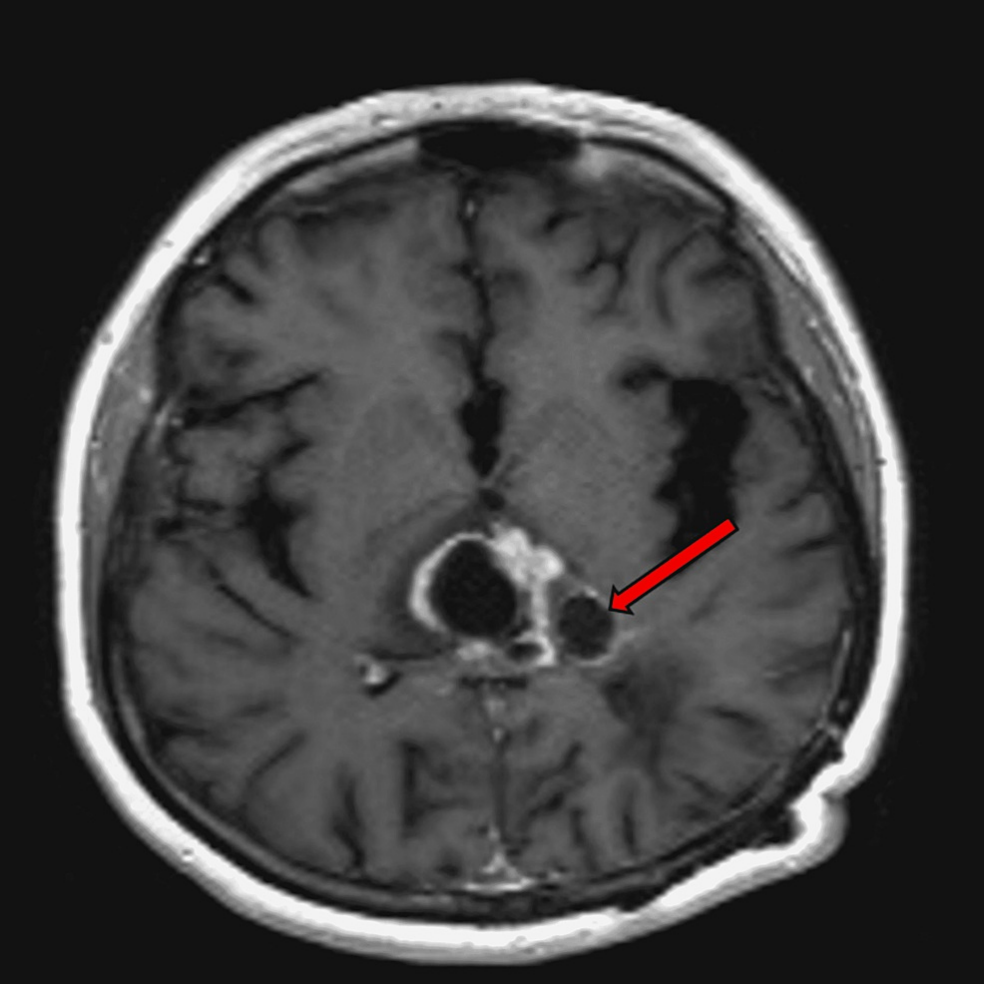
Tumor location in the corpus callosum

A 31-year-old woman presented with nausea, vomiting, headaches, and syncope in December 2017. She underwent a biopsy and subsequently she underwent resection after one week of biopsy. She received an accelerated course of three weeks of chemoradiation with concurrent temozolomide, and after one month, she had three shunts placed for hydrocephalus within eight months. She experienced tumor progression in April 2018 but could not receive salvage chemotherapy due to concurrent shunt infections. Her shunts were replaced through October 2018 at which time her tumor which was in the corpus callosum appeared to shrink on her November 2018 MRI (Figure 2). Tumor progression was noted in January 2019, and she underwent gamma knife receiving 19 Gy.

Her pathology report showed a neurofilament and immunostaining highlighted infiltrative growth by tumor. Epithelial membrane antigen (EMA) showed focal reactivity, and IDH1 performed was negative. ATRX showed loss of expression in tumor cells. The case was reviewed, and she underwent a biopsy which showed findings consistent with a diffuse midline glioma, H3K27M mutant (WHO grade IV), and Fluorescence In-Situ Hybridization (FISH), and unmethylated MGMT was observed. 

In July 2019, tumor progression was noted, and she received five cycles of bevacizumab approximately every 21 days. She received five doses of bevacizumab, and she was clinically improved and was radiographically stabilized until her latest brain MRI in November 2019 showed tumor progression. She was referred for an evaluation for eligibility for the expanded access clinical trial for ONC201 and, having met eligibility criteria, enrolled in the study in January 2020. She was reviewed in February 2020, one month after the initiation of ONC201, and her MRI images were reviewed and compared to prior images. The images show a corpus callosal and posterior cystic mass. The images did not meet the criteria for progression and were therefore deemed as stable disease (Table 2).

**Table 2:** Summary of adverse events reported in all three cases LDH: lactate dehydrogenase; ALT: alanine transaminase

		Case 1	Case 2	Case 3
Serious adverse effects related to medical history				
Nervous system disorders	Headache			x
	Right-sided pain			x
	Poor memory		x	x
	Slowness of thinking		x	
	Trouble speaking		x	
	Hemiparesis			x
	Ataxia	x	x	x
	Seizures		x	
	Poor appetite		x	
	Spinal cord compression	x		
Gastrointestinal disorders	Constipation		x	
	Hemorrhoids		x	
	Bowel incontinence	x		
General disorders	Anxiety			x
	Poor sleep			x
	Heat intolerance	x		
	Fatigue	x	x	
	Dizziness		x	
	Personality changes		x	
Musculoskeletal and connective tissue disorders	Back pain	x		
	Joint stiffness			x
	Neck stiffness		x	
	Joint pain			x
Urinary disorders	Urinary incontinence	x		
Eye disorders	Diplopia		x	x
	Blurry vision		x	
	Poor vision			x
Respiratory disorder	Thoracic disorders			
Serious adverse event not likely related to study				
Elevated calcium (grade 1)				x
Serious adverse event possibly related to study intervention				
Ankle swelling (grade I)		x		
Elevated LDH (grade I)			x	
Elevated magnesium (grade I)			x	
Elevated ALT (grade I)			x	
Leukocytosis (grade I)			x	

Case 3

**Figure 3:**
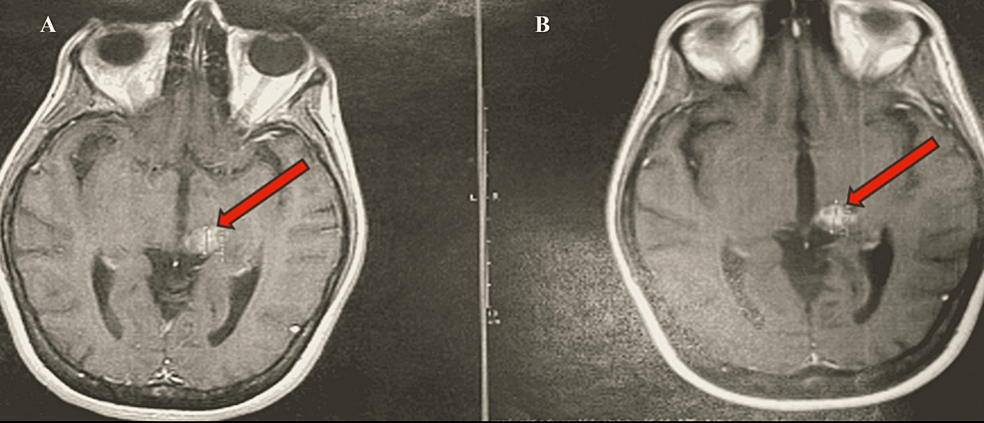
Left thalamic enhancing mass lesion with non-enhancing contrast CT measuring baseline measurement 1.28 cm × 2.06 cm × 2.84 cm (A) and cycle 2 measurement 1.6 cm × 1.6 cm × 2.56 cm (B)

A 44-year-old woman experienced right-sided numbness beginning in December 2017 that slowly progressed to the weakness of right arm and leg by January 2018. She was diagnosed with a glioblastoma in January 2018 after undergoing a stereotactic biopsy of a thalamic lesion and her tumor was notable for the H3K27M mutation, IDH1 negative, MGMT methylated (Figures 3A, 3B). She underwent concurrent chemoradiation with temozolomide in February 2018 receiving 30 fractions. Her radiology report showed left thalamic enhancing mass lesion with non-enhancing component (Figures 3A, 3B).

Her MRIs of September 2019, December 2019, and January 2020 showed gradual progression. The patient wished to discuss the ONC201 expanded access trial with her treating oncologist. The patient had significant right-sided paresthesia and allodynia. She was enrolled in the ON201 study and initiated on ONC201 625 mg (five caps) weekly. She did not experience side effects attributable to ONC201. Her pre- and post-MRI findings are highlighted in Figures 3A, 3B.

## Discussion

ONC201 causes expression and promotion of anti-tumor activity in cancer cells by inducing the tumor necrosis factor group of ligands (TRAIL) [29-31]. TRAIL bind to receptors DR4 and DR5, also known as death receptors, activates these receptors by paracrine or autocrine mechanisms and starts the process of programmed death of tumor cells [7]. ONC201 causes the dephosphorylation process of Foxo3a by inducing the inhibition of both ERK and Akt. This leads to the dislocation of Foxo3a into the nucleus from cytoplasmic region. Foxo3a causes upregulation of gene transcription by combining with TRAIL promoter [7]. There are two separate pathways reported by different research groups that the cell death process by cell stress mechanism is not dependent on TRAIL transcription. The most common example was presented in a study where ONC201 was not dependent on either Foxo3a-based transcription or caspase-8 activation [1,25].

All three patients presented in this case study had glioblastoma with varying tumor locations which includes thalamus, brainstem, and corpus callosum (Table 1). Improved survival rates were indicated by MGMT promoter methylation although they all had IDH wild type. MGMT promoter methylation was reported in two of three patients and IDH negative status in all patients (Table 1). While in other studies, this trend varies considerably [32], MGMT promoter methylation was highly associated with improved survival [33]. Other studies also support the fact that IDH mutation appeared highly prevalent in long-term survival (LTS) and is associated with survival [34-36].

In the same manner, telomerase reverse transcriptase (TERT) is a negative prognostic marker in a few studies but not all. Such factors are not absolute determinants of benefits or risk but may be studied in terms of improving or worsening the treatment outcome. A study using a mouse model showed that the ATRX gene provides a pathway for normal cell development so its loss may create chances of possible DNA damage causing breakdown of DNA strands [37]. Our research presented ATRX detection in all three patients and suggests that ONC201 also plays a role in the prevention of DNA damage by preserving ATRX gene function [37,38].

Median change from baseline KPS observed in our results appeared to be ≤20% decrease while other studies' results are also comparable for KPS values where baseline KPS reduction was not more than 20% [39]. Safety and reliability are also evident from the fact that there were no serious adverse events reported in any of these cases after the use of ONC201. The reported adverse events were of grades I-II (mild to moderate) which indicate a good safety profile when compared to other available therapies [2,40,41]. A limitation of our study is the few number of participants involved in it.

## Conclusions

Based on our measured parameters, ONC201 has demonstrated safety and efficacy in subjects receiving the study drug against H3K27M-mutant malignant glioma. Tolerability profile was remarkable for the study drug while safety is evident from the fact that adverse events recorded were mild to minimal. There were also no abnormal radiographic responses recorded from the use of ONC201. Future analyses are needed in larger groups of patients with multiple varying factors.
